# NOX4 inhibition promotes the remodeling of dystrophic muscle

**DOI:** 10.1172/jci.insight.158316

**Published:** 2022-10-24

**Authors:** David W. Hammers

**Affiliations:** 1 Department of Pharmacology & Therapeutics and; 2 Myology Institute, University of Florida College of Medicine, Gainesville, Florida, USA.

**Keywords:** Muscle Biology, Therapeutics, Fibrosis, Neuromuscular disease, Skeletal muscle

## Abstract

The muscular dystrophies (MDs) are genetic muscle diseases that result in progressive muscle degeneration followed by the fibrotic replacement of affected muscles as regenerative processes fail. Therapeutics that specifically address the fibrosis and failed regeneration associated with MDs represent a major unmet clinical need for MD patients, particularly those with advanced-stage disease progression. The current study investigated targeting NAD(P)H oxidase 4 (NOX4) as a potential strategy to reduce fibrosis and promote regeneration in disease-burdened muscle that models Duchenne muscular dystrophy (DMD). NOX4 was elevated in the muscles of dystrophic mice and DMD patients, localizing primarily to interstitial cells located between muscle fibers. Genetic and pharmacological targeting of NOX4 significantly reduced fibrosis in dystrophic respiratory and limb muscles. Mechanistically, NOX4 targeting decreased the number of fibrosis-depositing cells (myofibroblasts) and restored the number of muscle-specific stem cells (satellite cells) localized to their physiological niche, thereby rejuvenating muscle regeneration. Furthermore, acute inhibition of NOX4 was sufficient to induce apoptotic clearing of myofibroblasts within dystrophic muscle. These data indicate that targeting NOX4 is an effective strategy to promote the beneficial remodeling of disease-burdened muscle representative of DMD and, potentially, other MDs and muscle pathologies.

## Graphical abstract

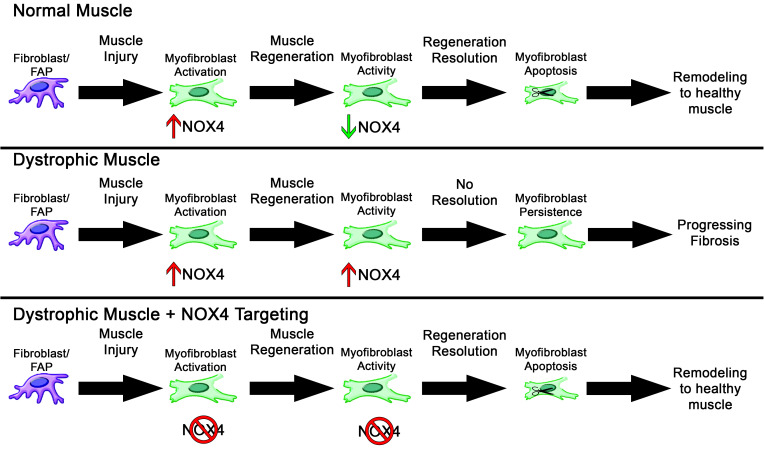

## Introduction

Muscular dystrophies (MDs) are genetic muscle diseases characterized by progressive skeletal muscle degeneration and replacement of functional musculature with an aberrant fibrotic extracellular matrix (ECM). Duchenne MD (DMD), the most prevalent of MDs, is a fatal, childhood-onset X-linked disease that affects approximately 1:5000 males (1). DMD is caused by mutations in the *DMD* gene resulting in complete loss of dystrophin (2), a protein that stabilizes the sarcolemma during contractile activity by providing a link between the muscle cytoskeleton and surrounding ECM (3). Patients with this devastating disease typically progress to loss of ambulation within the second decade of life and death by the age of 30. During the course of disease progression, DMD patients exhibit a profound expansion of a fibrotic and fatty ECM as muscle fibers are lost (4). Therapeutics capable of slowing or reversing this ECM expansion are a major unmet clinical need, particularly for disease management of older DMD patients.

The identification of treatments that effectively improve dystrophic muscle has been largely hindered by limited knowledge of the cellular mechanisms responsible for the development of muscle fibrosis and impairment of muscle regeneration associated with the disease. Under normal conditions, skeletal muscle displays robust regeneration following injury, which is largely mediated by muscle-resident stem cells, known as satellite cells (5, 6). The process of muscle regeneration also requires an array of chemical and physical cues provided by immune cells, myogenic cells, fibroblasts, and other cellular populations (7–9). These factors are temporally orchestrated to form new muscle that is accommodated with adequate vasculature, innervation, and ECM structural support, leading to resolution of the injury response and return to homeostasis (5, 7, 10). Indeed, perturbations to any of these components contributes to maladaptive muscle regeneration (7, 9, 11, 12). In dystrophic muscle, the continuous and asynchronous combination of degeneration and regenerative processes within the same muscle disrupts the timing of these events (13). This ultimately leads to regenerative impairments and progressing fibrosis that characterize the disease burden of late-stage dystrophic muscle.

The origins of fibrotic pathologies are linked to the activation of specialized ECM-secreting cells, known as myofibroblasts. During the injury repair process, myofibroblasts differentiate from resident fibroblasts to replace damaged ECM. They then undergo clearance, typically via apoptosis, at the resolution stages of regeneration (reviewed in ref. 14). In the event myofibroblast clearance is disrupted, however, ECM deposition continues and results in pathological fibrosis. Therefore, strategies to prevent and/or reverse this persistence of myofibroblasts offer great promise for the treatment of fibrotic diseases.

Excessive reactive oxygen species (ROS) generation has been implicated in the mechanisms leading to myofibroblast activation and development of fibrosis, particularly those produced by the NAD(P)H oxidase (NOX) family of enzymes (15). NOX4, specifically, has been identified as a promising antifibrotic target in several organs, including lung, kidney, liver, and heart (reviewed in ref. 16). While elevated *Nox4* expression has been noted in dystrophic mouse hearts (17), a causative role for NOX4 has not been previously investigated in skeletal muscle fibrosis. Using a previously published transcriptomic data set (18), NOX4 was found upregulated in diseased muscle of D2.*mdx* mice, a severe mouse model of DMD (19–21). The current study sought to determine whether targeting NOX4 is an effective strategy to prevent fibrosis and enhance regeneration in dystrophic muscle. Herein, it is shown that NOX4 localizes primarily to interstitial cells of dystrophic muscle, including myofibroblasts. The targeting of NOX4 both by genetic ablation and pharmacological inhibition promotes the beneficial remodeling of diseased muscle by reducing muscle fibrosis. Importantly, NOX4 targeting reduces myofibroblasts within disease-burdened muscle, restores the localization of satellite cells to their physiological niche, and increases evidence of muscle regeneration. These data implicate NOX4 in the development of MD-associated skeletal muscle pathology, and demonstrate that targeting NOX4 is an effective strategy to promote beneficial remodeling of dystrophic muscle.

## Results

### NOX4 is increased in the interstitium of dystrophic muscle.

**Figure 1:**
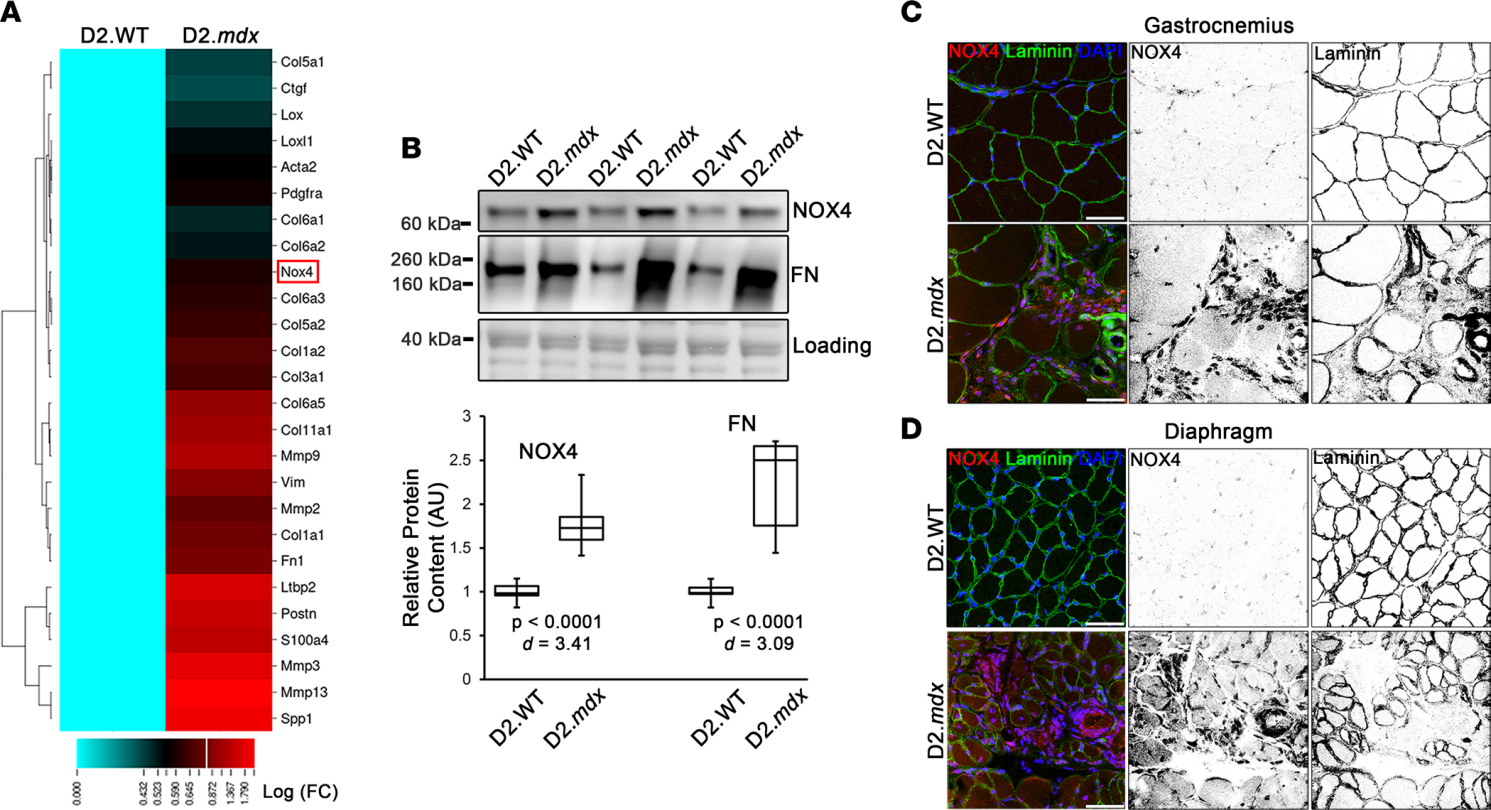
NOX4 is increased in dystrophic skeletal muscle. (**A**) Fibrosis-associated genes differentially expressed in 4-month-old male wild-type DBA/2J (D2.WT) and dystrophic D2.*mdx* quadriceps muscles were queried from transcriptomic data (*P* < 0.05; depicted as log[fold change]; published in ref. 18). *Nox4* is highlighted by the red box. (**B**) Immunoblotting verifies differential content of NOX4 and fibronectin (FN) protein levels in quadriceps lysates from 4-month-old male D2.WT and D2.*mdx* mice (*n* = 7). Immunofluorescent detection of NOX4 in the (**C**) gastrocnemius and (**D**) diaphragm muscles of D2.WT and D2.*mdx* mice reveals that NOX4 is primarily localized to interstitial cells of dystrophic muscle. Scale bars: 100 μm. Data are presented as box-and-whisker plots, with whiskers representing minimum and maximum values, and were analyzed using unpaired, 2-tailed Welch’s *t* test (α = 0.05; effect size is presented as Cohen’s *d*).

The heightened skeletal muscle fibrosis of D2.*mdx* mice makes this emerging mouse model of DMD better suited to investigate mechanisms contributing to muscle fibrosis than *mdx* mice on C57-based genetic backgrounds (19). To identify potential gene targets that may be exploited as antifibrotic therapies, a previously published transcriptomic data set (18) was queried for ECM/fibrosis-associated genes that are significantly upregulated in D2.*mdx* quadriceps muscle, relative to wild-type DBA/2J (D2.WT) values (*P* < 0.05). Of 26 genes identified (Figure 1A), the majority consisted of ECM components, including 10 isoforms of collagen, 4 members of matrix metallopeptidases (MMPs; a class of matrix remodeling enzymes), the collagen-crosslinking enzymes *Lox* and *Loxl1*, and other ECM-related genes, including *Fn1* (fibronectin), *Ltbp2*, *Postn* (periostin), and *Spp1* (osteopontin). Identified fibroblast-lineage markers include *Pdgfra* (platelet-derived growth factor receptor α [PDGFRα]), *Ctgf*, *Vim* (vimentin), *S100a4*, and *Acta2* (α-smooth muscle actin [αSMA]).

**Figure 2:**
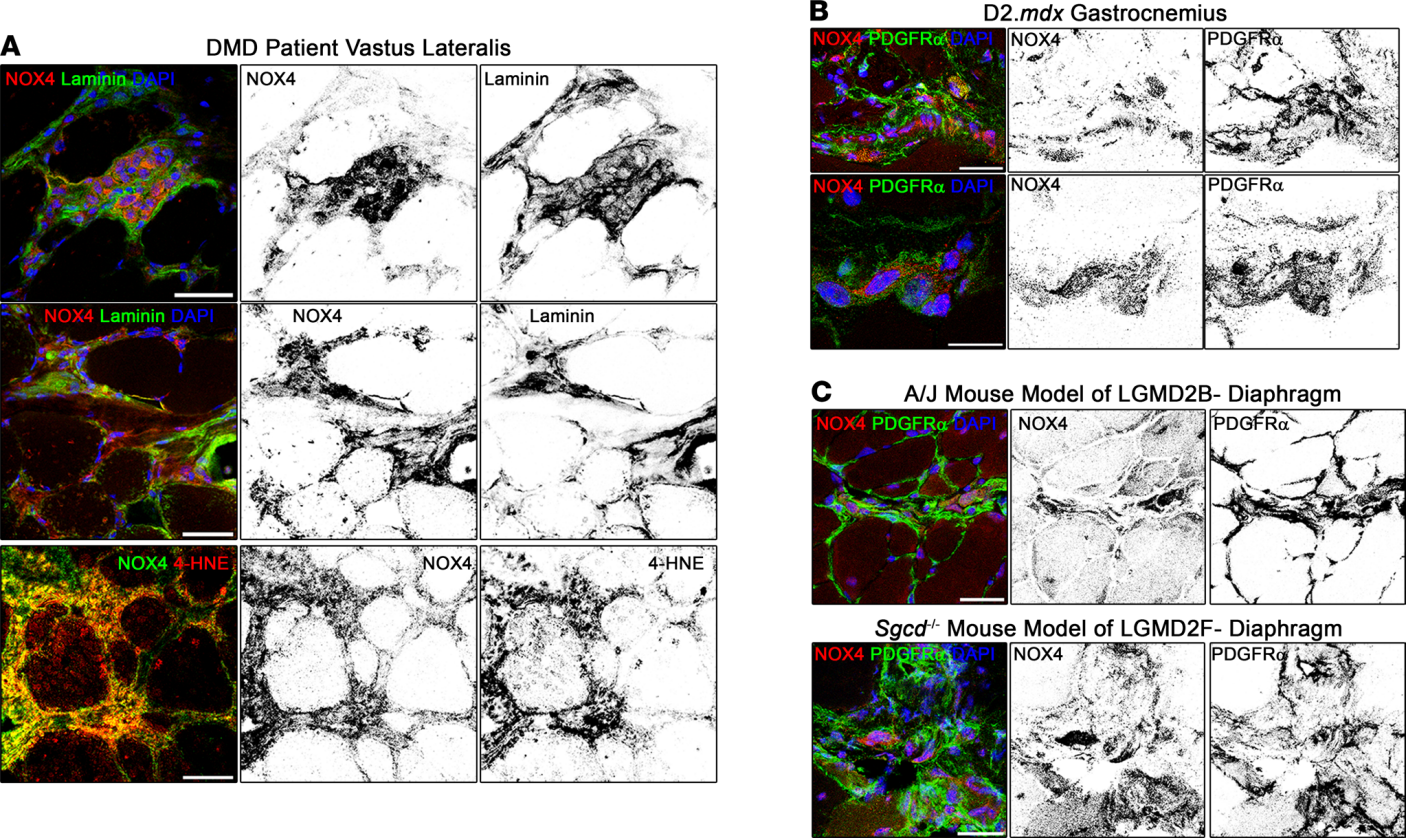
Interstitial NOX4 colocalizes with isoprostane generation in DMD patient muscle. (**A**) Immunofluorescence analysis of DMD patient muscle sections reveals that interstitial NOX4 localization occurs in human dystrophic muscle and colocalizes with areas enriched with 4-hydroxynonenal (4-HNE), a product of lipid peroxidation. Interstitial NOX4 localizes largely with fibroblastic cells labeled by PDGFRα in muscles of (**B**) the D2.*mdx* mouse model of DMD, as well as (**C**) those of other muscular dystrophy models, including 9-month-old A/J mice (model of LGMD2B) and 3-month-old *Sgcd^–/–^* mice (model of LGMD2F). Scale bars: 50 μm.

An interesting find among this query was the increased expression of *Nox4*, which encodes the intracellular ROS-generating enzyme, NOX4. NOX4 has been identified as an antifibrosis target in several tissue types (22–29), but has not previously been associated with skeletal muscle fibrosis. Importantly, safe clinical-stage small-molecule inhibitors of this enzyme have been developed (30). Immunoblotting confirmed that NOX4 protein was elevated in D2.*mdx* skeletal muscle (Figure 1B), which was localized at high concentrations in the interstitial regions between muscle fibers of D2.*mdx* mice, as shown in the gastrocnemius (Figure 1C) and diaphragm (Figure 1D). This interstitial localization of NOX4 was also observed in the muscles of DMD patients (Figure 2A). Importantly, NOX4 colocalizes to regions of intense immunoreactivity for 4-hydroxynonenal (4-HNE; Figure 2A), a product of damaging lipid peroxidation that is associated with fibrotic pathologies (31, 32). This indicates that substantial ROS generation occurred in these NOX4-enriched regions of DMD muscle. The NOX4-expressing cells observed in D2.*mdx* muscle also stained positive for PDGFRα (Figure 2B), a marker of fibroblastic mesenchymal cells, including fibroadipogenic progenitors (FAPs), fibroblasts, and myofibroblasts (33–36). NOX4-expressing PDGFRα^+^ cells were also found in the muscles of mice that model limb-girdle MD 2B (LGMD2B) (dysferlinopathy) and LGMD2F (δ-sarcoglycan deficiency) (Figure 2C); therefore, these observations are not restricted to dystrophin-deficient muscle.

In agreement with the identification of myofibroblast NOX4 contributing to human fibrotic diseases, including idiopathic pulmonary fibrosis (25), robust NOX4 immunoreactivity was found localized to myofibroblasts in DMD patient muscle (Supplemental Figure 1A; supplemental material available online with this article; https://doi.org/10.1172/jci.insight.158316DS1), identified by costaining for αSMA and vimentin (37). While murine myofibroblasts could be similarly identified in D2.*mdx* muscle sections by costaining for PDGFRα, vimentin, and αSMA (Supplemental Figure 1B), antibody incompatibility prevented the clear labeling of NOX4 specifically in myofibroblasts using tissue immunofluorescence. However, isolation of PDGFRα^+^ cells from D2.*mdx* muscle revealed strong NOX4 staining in myofibroblasts (Supplemental Figure 1C), which are distinguishable from fibroblasts based on morphology and immunoreactivity for Postn and αSMA. This evidence supports the hypothesis that NOX4 is involved in the development of MD-associated muscle fibrosis and that NOX4 targeting may be an efficacious strategy to prevent the fibrotic replacement of muscle associated with these diseases.

### NOX4 targeting reduces fibrosis in dystrophic muscle.

The role of NOX4 in the development of muscle fibrosis was directly tested by generating a *Nox4*-knockout mouse line on the D2.*mdx* background (Nox4^KO^:*mdx*). Wild-type littermates of this line (Nox4^WT^:*mdx*) were indistinguishable from mice of the D2.*mdx* colony and exhibited NOX4 staining in the muscle interstitium, whereas Nox4^KO^:*mdx* mice showed no NOX4 immunoreactivity (Supplemental Figure 2A). At 3 months of age, when D2.*mdx* mice begin to show fibrosis during recovery from widespread muscle degeneration (19), Nox4^WT^:*mdx* and Nox4^KO^:*mdx* littermates showed no difference in muscle fibrosis, as assessed by histological evaluation using picrosirius red staining of the diaphragm and gastrocnemius (Supplemental Figure 2B). Therefore, NOX4 ablation does not appear to (a) prevent the degenerative phase of D2.*mdx* muscle pathology and, importantly, (b) adversely affect ECM production associated with tissue repair from injury.

**Figure 3:**
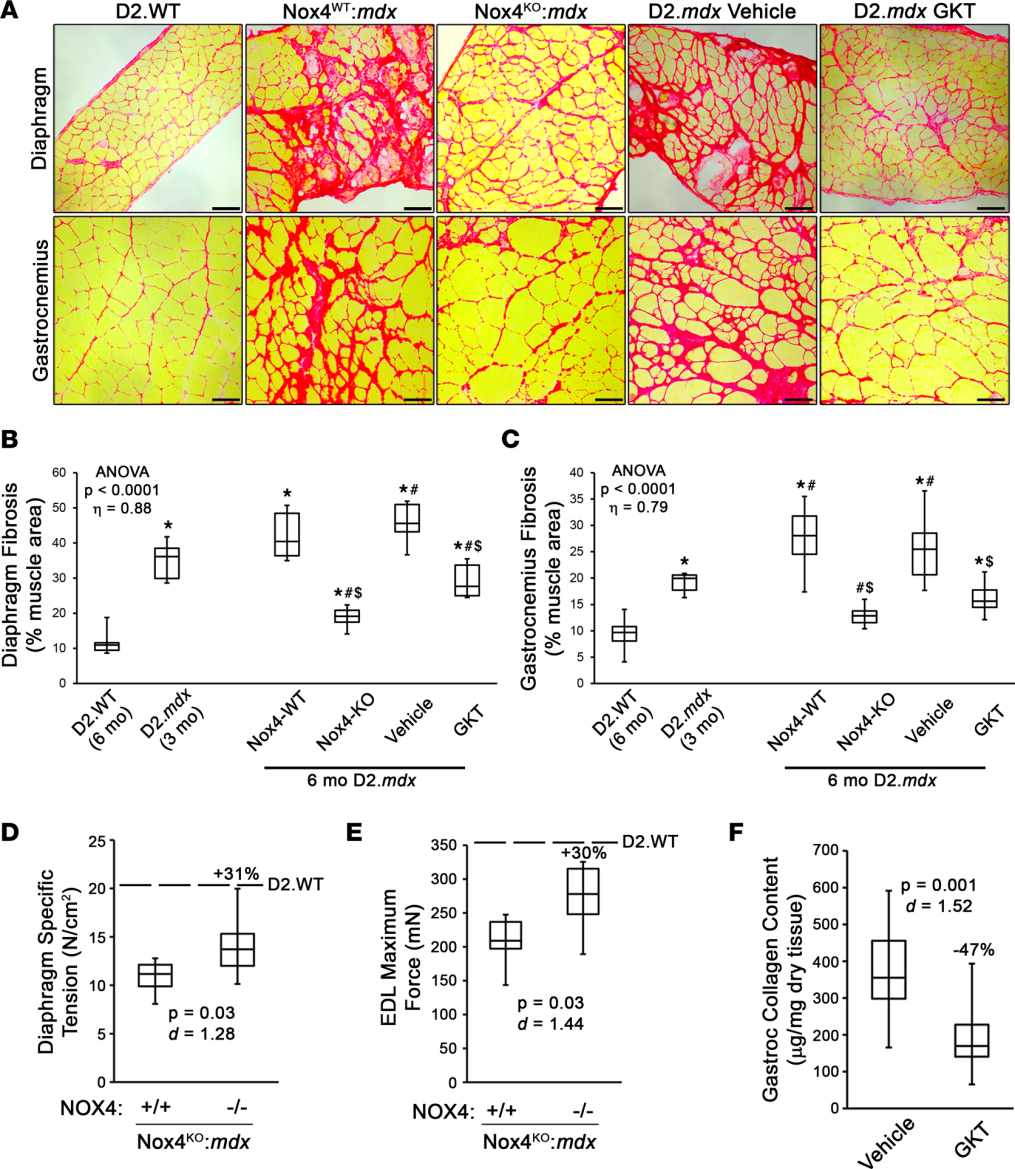
Targeting NOX4 reduces fibrosis in dystrophic muscle. (**A**) Representative picrosirius red staining and image quantification of (**B**) diaphragm and (**C**) gastrocnemius muscles from 6-month-old D2.WT (*n* = 6), NOX4 wild-type (NOX4-WT), and knockout (NOX4-KO) littermates from a NOX4-KO line generated on the D2.*mdx* background (Nox4^KO^:*mdx*; *n* = 12), and D2.*mdx* mice that received vehicle or GK831 (GKT) treatments beginning at 3 months of age (*n* = 12–14). Fibrosis quantifications for 3-month-old D2.*mdx* mice are included to show baseline values for GKT treatment groups. Scale bars: 100 μm. Muscle function was assessed for the (**D**) diaphragm and (**E**) extensor digitorum longus (EDL) muscles of NOX4-WT (+/+) and NOX4-KO (–/–) littermates of the Nox4^KO^:*mdx* mouse line (*n* = 6). (**F**) Biochemical quantification of collagen content was performed on gastrocnemius muscles of vehicle- and GKT-treated D2.*mdx* mice (*n* = 9–12). Data are presented as box-and-whisker plots, with whiskers representing minimum and maximum values. In **D**–**F**, percentage values indicate the difference between mean values of the 2 groups. Data were analyzed using (**B** and **C**) ANOVA followed by Tukey’s post hoc test (α = 0.05; effect size is reported as *η*^2^) or (**D**–**F**) unpaired, 2-tailed Welch’s *t* test (α = 0.05; effect size is reported as Cohen’s *d*). “ANOVA *P* < 0.0001” in **B** and **C** refers to the significance level of the entire data set, consisting of D2.WT, D2.mdx (3 mo), Nox4-WT, Nox4-KO, vehicle, and GKT groups. *P < 0.05 vs. D2.WT values; ^#^*P* < 0.05 vs. 3-month-old D2.*mdx* values; ^$^*P* < 0.05 vs. respective control group values.

At 6 months of age, when D2.*mdx* muscle displays robust fibrotic progression independently of reparative activities (19), Nox4^KO^:*mdx* diaphragms and gastrocnemius muscles exhibited significantly less fibrosis than those of the Nox4^WT^:*mdx* littermates (Figure 3, A–C). While Nox4^WT^:*mdx* muscles showed the expected increase in fibrosis from 3 months to 6 months, Nox4^KO^:*mdx* muscles actually reduced muscle fibrosis during this timeframe. Thus, loss of NOX4 prevents the progressive accumulation of fibrosis in D2.*mdx* skeletal muscle, possibly by facilitating resolution of the postinjury response. NOX4-ablated diaphragms and extensor digitorum longus (EDL) muscles also exhibited significantly increased muscle function over those of Nox4^WT^:*mdx* mice (Figure 3, D and E), demonstrating that functional muscle is better maintained as fibrosis is reduced. In addition to these skeletal muscle benefits, the hearts of Nox4^KO^:*mdx* mice had less left ventricular fibrosis than those from their Nox4^WT^ littermates (Supplemental Figure 2C).

While it is hypothesized that the benefits incurred by NOX4 ablation are largely mediated by targeting the interstitial environment, myofiber-specific contributions to these improvements are also possible and, thus, were investigated. In agreement with the lack of protection from degeneration in the early phase of disease progression, NOX4 ablation did not affect sarcolemmal utrophin content (Supplemental Figure 3A), which can functionally compensate for loss of dystrophin when upregulated (38). NOX4 has been shown to regulate muscle metabolism following exercise (39) and enhance vascularity during cardiac stress (40). In the context of MD, however, no evidence of changes in mitochondrial content, as evaluated through immunoblotting for the mitochondrial proteins succinate dehydrogenase A and heat shock protein 60 (Supplemental Figure 3B), or blood vessel density, as evaluated by CD31^+^ staining (Supplemental Figure 3C), were observed as a result of NOX4 ablation. NOX4-ablated gastrocnemius muscles did, interestingly, exhibit a slightly reduced proportion of type I (slow oxidative) muscle fibers compared with Nox4^WT^:*mdx* littermates (Supplemental Figure 3D). It is unclear at this time whether this was due to intrinsic mechanisms regulating muscle fiber type specification or indicative that loss of fast glycolytic (type IIX/B) fibers differs between the genotypes during the course of disease progression. In further support that NOX4 ablation affects the interstitial environment of dystrophic muscle, interstitial 4-HNE (defined using the methodology depicted in Supplemental Figure 4A) was significantly reduced in Nox4^KO^:*mdx* muscle (Supplemental Figure 4B). This confirms that NOX4 ablation reduces interstitial ROS generation, which is linked to a substantial reduction in muscle fibrosis. The presence of 4-HNE staining in the muscle fibers of Nox4^KO^:*mdx* mice suggests that other sources of ROS, such as NOX2 (41, 42), contribute to lipid peroxidation occurring inside dystrophic muscle fibers.

Small-molecule drugs capable of inhibiting NOX4 have been developed and safely implemented in the clinic (16, 30). In order to test the potential translational value of NOX4 targeting with such compounds, a 3-month trial with the NOX1/4 inhibitor GKT831 (22, 30, 43) was also performed. GKT831 reduced D2.*mdx* muscle fibrosis to a comparable extent as NOX4 ablation, when evaluated at 6 months of age (Figure 3, A–C, and F). Therefore, pharmacological inhibition of NOX4 phenocopies the antifibrotic efficacy of NOX4 ablation. Collectively, these data indicate that targeting NOX4 is an efficacious means to control the fibrotic replacement of dystrophic muscle in the D2.*mdx* preclinical model of DMD.

### NOX4 targeting promotes dystrophic muscle remodeling through clearance of myofibroblasts.

**Figure 4:**
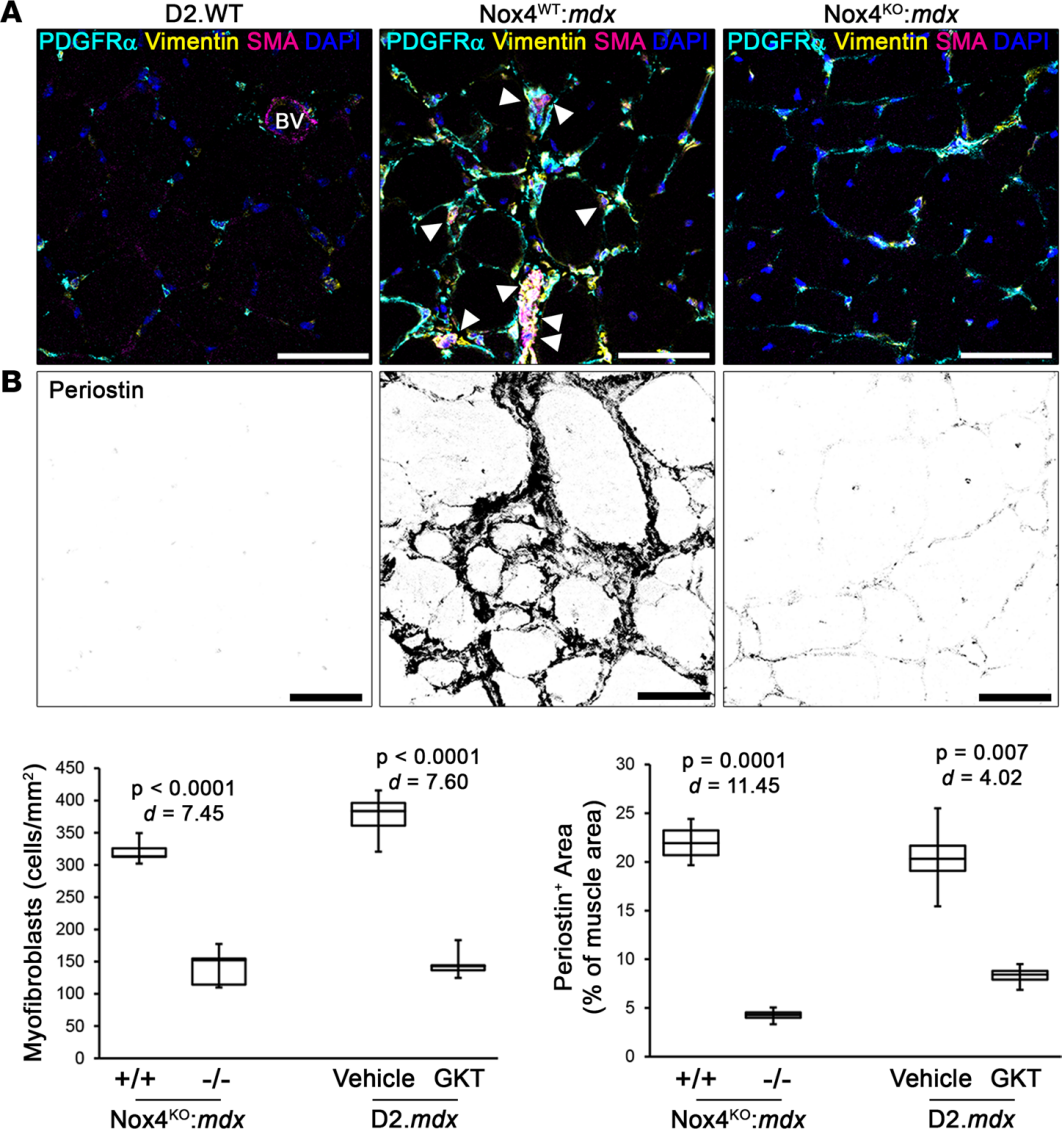
NOX4 targeting reduces myofibroblasts in dystrophic muscle. Myofibroblast numbers were assessed in gastrocnemius muscles from 6-month-old Nox4^WT^:*mdx* (+/+), Nox4^KO^:*mdx* (–/–), and D2.*mdx* mice receiving vehicle or GKT831 (GKT) treatments. Representative immunofluorescence images and quantifications are shown for (**A**) myofibroblast prevalence, as determined by colabeling for PDGFRα, vimentin, and αSMA (arrows mark myofibroblasts; BV = blood vessel; *n* = 6–12), and (**B**) muscle periostin content (*n* = 6–12). Data are presented as box-and-whisker plots, with whiskers representing minimum and maximum values, and were analyzed using unpaired, 2-tailed Welch’s *t* test (α = 0.05; effect size is reported as Cohen’s *d*). Scale bars: 50 μm.

Myofibroblasts actively secrete ECM in tissues during repair and disease (14), and NOX4 is expressed by myofibroblasts in fibrotic lungs (25) and dystrophic muscle (Supplemental Figure 1, A and C). Therefore, it is reasonable to suspect that NOX4 targeting exerts its antifibrotic effects by targeting myofibroblasts. In agreement, myofibroblast numbers were significantly reduced by both NOX4 ablation and GKT831 treatment (Figure 4). Additionally, these NOX4-targeting strategies reduced the amount of Postn (Figure 4), an ECM component specifically expressed and secreted by myofibroblasts in muscle tissues (44). The loss of NOX4 also affected other fibroblastic populations in dystrophic muscle, as total numbers of PDGFRα^+^ cells were reduced in Nox4^KO^:*mdx* muscles (Supplemental Figure 4C), whereas the proportion of stem cell antigen 1–positive (Sca-1^+^) FAPs within the PDGFRα^+^ population remained unchanged by loss of NOX4. These data indicate that the antifibrotic efficacy of NOX4 inhibition in dystrophic muscle is due to clearance of fibroblastic cells, particularly myofibroblasts, thereby removing the source of pathological ECM deposition.

**Figure 5:**
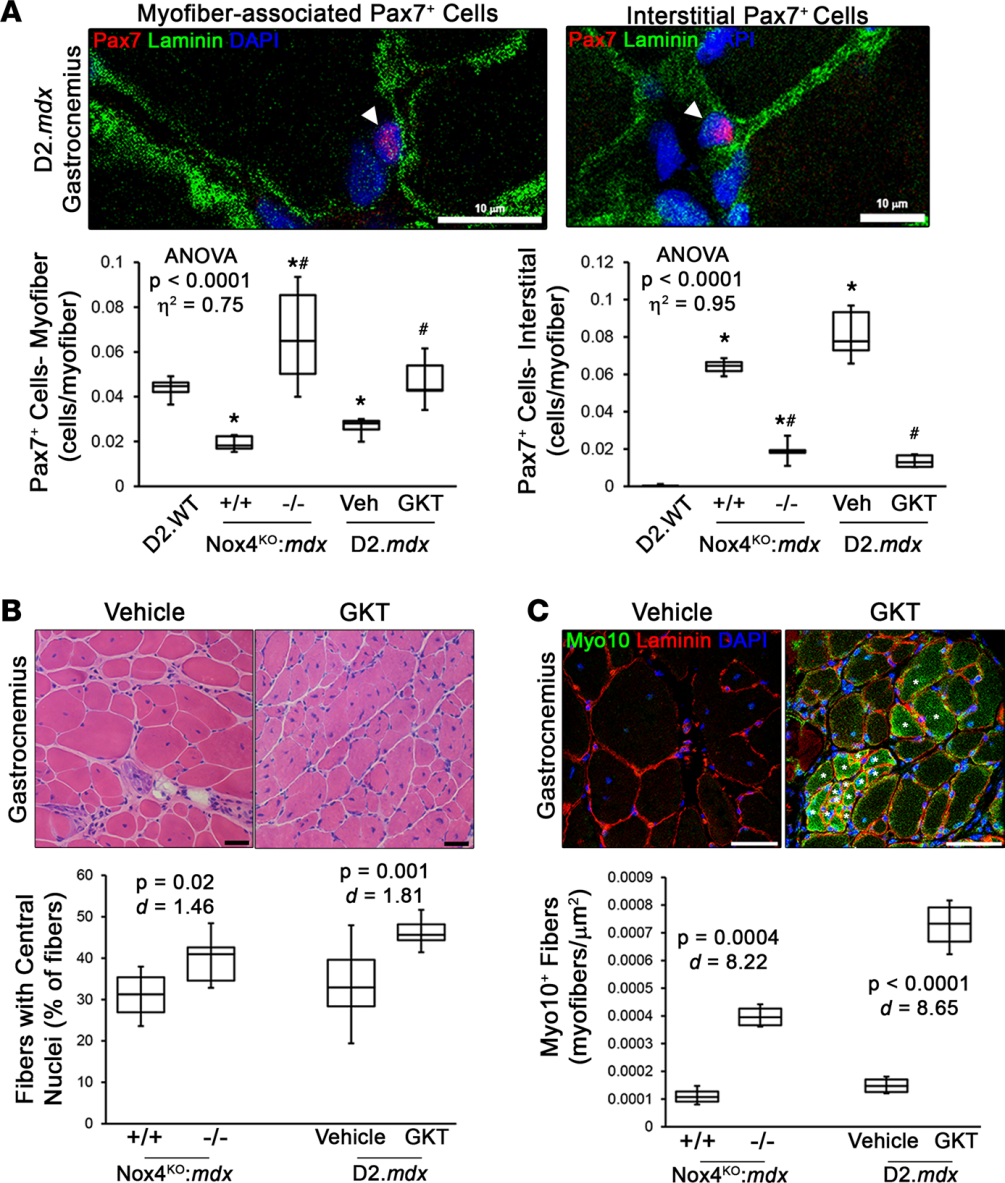
Muscle regeneration is improved by NOX4 targeting. Gastrocnemius muscles from 6-month-old D2.WT, Nox4^WT^:*mdx* (+/+), Nox4^KO^:*mdx* (–/–), and D2.*mdx* mice receiving vehicle (Veh) or GKT831 (GKT) treatments were assessed for indices of muscle regeneration. (**A**) Pax7 immunofluorescence was performed to assess satellite cell numbers. Populations of myofiber-associated and interstitial Pax7^+^ cells were identified and affected by NOX4 targeting, as displayed by representative images and population quantifications (arrows mark Pax7^+^ cells; *n* = 6–12). Evidence of restarted muscle regeneration by NOX4 targeting was also found, as shown by number of fibers exhibiting (**B**) centrally located nuclei (*n* = 6–12) and (**C**) staining for myosin X (Myo10; stars indicate fibers marked as recently regenerated; *n* = 6–12). Data are presented as box-and-whisker plots, with whiskers representing minimum and maximum values, and were analyzed using (**A**) ANOVA followed by Tukey’s post hoc test (α = 0.05; effect size is reported as *η*^2^; *P < 0.05 vs. D2.WT values; ^#^*P* < 0.05 vs. respective control group values) or (**B** and **C**) unpaired, 2-tailed Welch’s *t* test (α = 0.05; effect size is reported as Cohen’s *d*). “ANOVA *P* < 0.0001” in **A** refers to the significance level of the entire data set, consisting of D2.WT, D2.mdx (3 mo), Nox4-WT, Nox4-KO, vehicle, and GKT groups. Scale bars: 10 μm (**A**) and 50 μm (**B** and **C**).

Evidence suggests that myofibroblasts inhibit muscle regeneration (45–47). In agreement with a suppressed regenerative capacity in disease-burdened D2.*mdx* muscle, Nox4^WT^:*mdx* and vehicle-treated gastrocnemius muscles exhibited significantly reduced numbers of Pax7^+^ satellite cells localized to their physiological niche between the sarcolemma and basal lamina of muscle fibers (Figure 5A). However, a substantial number of Pax7^+^ cells were found in the dystrophic muscle interstitium (Figure 5A), similar to what has been previously shown in DMD patient muscle (48) and murine cancer cachexia (49). In agreement with the beneficial remodeling of the dystrophic musculature by NOX4 targeting, Pax7^+^ cell localization was restored back to the periphery of muscle fibers with NOX4 ablation and GKT831 treatment (Figure 5A). Furthermore, this return of Pax7^+^ cells to their physiological niche coincided with increases in muscle fibers having central nuclei (Figure 5B) and expression of myosin X (Figure 5C), both indicators of recent muscle regeneration (10, 50). These data suggest NOX4 targeting, likely indirectly through the clearance of myofibroblasts, allows stalled satellite cells located in the muscle interstitium to reenter the myogenic program, participate in muscle regeneration, and reestablish their physiological niche, thereby facilitating the beneficial remodeling of dystrophic muscle by restoring muscle’s regenerative capacity.

**Figure 6:**
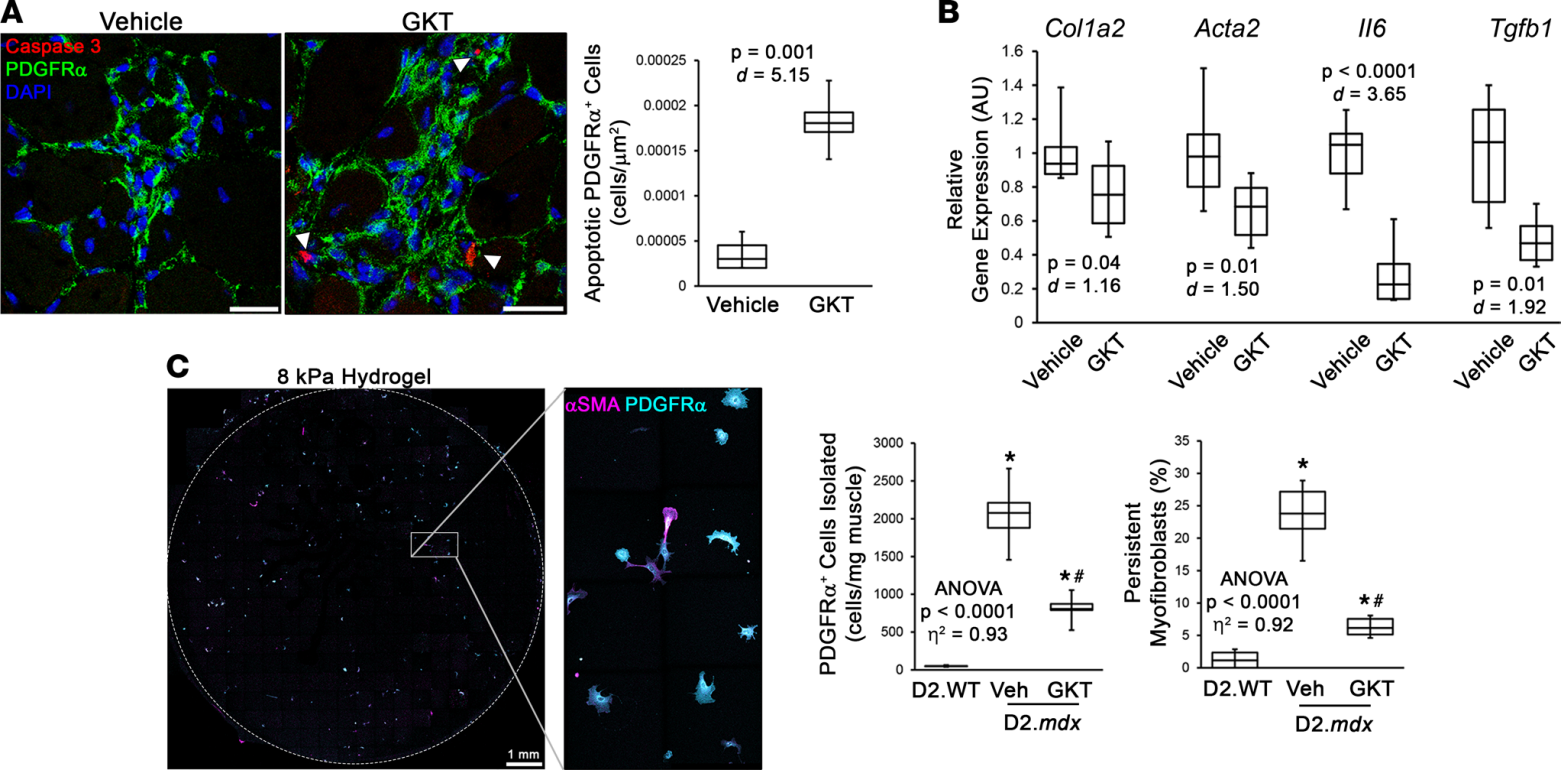
Short-term NOX4 inhibition induces myofibroblast apoptosis and reduces myofibroblast persistence. Six-month-old male D2.*mdx* mice were treated with vehicle or 60 mg/kg GKT831 (GKT) for 7 days. (**A**) Apoptosis of PDGFRα^+^ cells in vehicle- (Veh) and GKT-treated gastrocnemius muscles was assessed by immunofluorescent detection of active caspase-3 (*n* = 6). (**B**) Gene expression for *Col1a2*, *Acta2*, *Il6*, and *Tgfb1* was also measured in gastrocnemius muscles using real-time PCR (*n* = 6). (**C**) Myoblast persistence was assayed by culturing PDGFRα^+^ cells isolated from D2.WT, Veh-treated D2.*mdx*, and GKT-treated D2.*mdx* gastrocnemius muscles on 8 kPa hydrogels for 3 days, followed by quantification of αSMA^+^ cells via immunofluorescence (*n* = 6). Data are presented as box-and-whisker plots, with whiskers representing minimum and maximum values, and were analyzed using (**A** and **B**) unpaired, 2-tailed Welch’s *t* test (α = 0.05; effect size is reported as Cohen’s *d*) or (**C**) ANOVA followed by Tukey’s post hoc test (α = 0.05; effect size is reported as *η*^2^). “ANOVA *P* < 0.0001” in **C** refers to the significance level of the entire data set, consisting of D2.WT, D2.mdx (3 mo), Nox4-WT, Nox4-KO, vehicle, and GKT groups. *P < 0.05 vs. D2.WT values; ^#^*P* < 0.05 vs. Veh group values. Scale bars: 50 μm (**A**) and 1 mm (**C**).

These data depict a model whereby NOX4 targeting promotes the remodeling of dystrophic muscle via the clearance of myofibroblasts and alleviation of regeneration-inhibiting actions on satellite cells. Because the presumed mechanism attributed to myofibroblast clearance is by promoting their apoptosis (14), a short-term study was performed to investigate acute changes in muscle that result from NOX4 inhibition. Six-month-old D2.*mdx* mice, whose muscles exhibit substantial disease burden (19), received vehicle or GKT831 treatments for 7 days. Following this dosing period, the gastrocnemius muscles of GKT831-treated mice exhibited significantly more PDGFRα^+^ cells showing positive staining for active caspase-3 (Figure 6A), an indicator of apoptosis. This demonstrates that the cellular clearance achieved by NOX4 inhibition occurs, at least in part, via apoptosis. In agreement, muscles of GKT831-treated mice also exhibited decreased expression of the fibrosis/myofibroblast markers *Col1a2* and *Acta2*, as well as the profibrotic and antiregeneration markers *Il6* and *Tgfb1*, compared with those of vehicle-treated mice (Figure 6B). This evidence reinforces the hypothesis that NOX4 inhibition promotes the remodeling of dystrophic muscle by inducing apoptosis in a population of cells that possibly represents persistent myofibroblasts.

To determine whether NOX4 inhibition reduces persistent myofibroblasts in dystrophic muscle, an assay was developed utilizing the recently published insight that persistent myofibroblasts can be effectively distinguished from nonmyofibroblast and transient myofibroblast populations by culturing on soft surfaces (51). This assay was performed by isolating PDGFRα^+^ cells from the gastrocnemius muscles of 6-month-old D2.WT and D2.*mdx* mice that had received 7 days of treatment with vehicle or GKT831, and culturing them on soft (8 kPa stiffness) hydrogels for 3 days. Following this incubation period, the cells were stained for PDGFRα and αSMA, and persistent myofibroblasts were quantified as the percentage of αSMA^+^ cells remaining on the hydrogel (Figure 6C). As anticipated, both total PGDFRα^+^ cells isolated from the muscle and the percentage of αSMA^+^ cells persisting on the hydrogels were elevated in vehicle-treated D2.*mdx* samples, compared with those from D2.WT mice (Figure 6C). Consistent with the observations of apoptosis following this acute treatment protocol and the hypothesis that persistent myofibroblasts are a target of NOX4 inhibition, both of these measures were significantly reduced following GKT831 treatment (Figure 6C). These results indicate that persistent myofibroblasts in dystrophic muscle can be effectively targeted by NOX4 inhibition.

## Discussion

Therapeutics capable of remodeling the disease-burdened muscle of MD patients is a major unmet clinical need. This is particularly important for the treatment of older DMD patients who have undergone substantial replacement of muscle with pathological ECM. The current work identifies NOX4 as an efficacious target to promote the remodeling of dystrophic muscle by preventing fibrosis and enhancing regeneration. This was achieved using genetic ablation and, importantly, pharmacological inhibition with a clinical-stage drug. The efficacious effect of NOX4 targeting is primarily associated with a reduction in myofibroblasts in dystrophic muscle. This clearance of myofibroblasts appears to alleviate both the profibrotic and antiregenerative environment associated with disease-burdened muscle. These findings indicate that NOX4-targeting interventions represent potential remodeling therapeutics capable of improving the muscle disease state in DMD and other muscle diseases.

Myofibroblasts are specialized ECM-depositing cells that are activated to facilitate tissue repair; however, they contribute to tissue pathology in chronic diseases by producing excessive ECM, thereby causing progressive fibrosis (14). A major finding of the current work is that the beneficial remodeling incurred by NOX4 targeting involves myofibroblast clearance from the muscle. This clearance includes a significant reduction in myofibroblasts that exhibit features of persistence, specifically continued expression of αSMA following several days of culture on a soft surface (51). In agreement with these findings, NOX4 inhibition also results in the clearance of myofibroblasts from fibrotic lungs (25, 52). In dystrophic muscle, the clearing of myofibroblasts via NOX4 targeting appears to be a 2-hit approach to promote beneficial remodeling, as (a) the cellular source of fibrosis is removed from the system, and (b) negative regulation of regenerative efforts is alleviated, thereby allowing a stalled myogenic program to proceed.

The inhibitory effect of myofibroblasts on muscle regeneration may be mediated by multiple mechanisms. For instance, the myofibroblast-specific protein Postn can directly inhibit muscle regeneration (45), while Postn ablation enhances regeneration (16, 46). Similarly, Postn content in dystrophic muscle is drastically reduced by NOX4 targeting (Figure 4), which coincides with evidence of rejuvenated regeneration (Figure 5). Furthermore, myofibroblasts may also release/activate signaling molecules that impede satellite cell function, such as TGF-β (53, 54). In this study, many Pax7^+^ cells were found in the dystrophic muscle interstitium (Figure 5A), which were largely restored to a myofiber-associated localization upon the removal of myofibroblasts by NOX4 targeting. This suggests that myofibroblasts, whether by secreted factors or physical interactions, essentially halt the activity of satellite cells within dystrophic muscle, thus contributing to the regenerative decline associated with DMD. Importantly, this arrest of satellite cell activity appears to be reversible, as was previously shown in cancer cachexia (49), which allows active regeneration of dystrophic muscle to be restarted upon alleviation of these inhibitory signals.

Knowledge of the diversity and plasticity among different subpopulations/differentiation states of mesenchymal/fibroblastic cells located in the muscle interstitium, commonly referred to as FAPs, has been advanced by recent findings. The work of Leinroth et al., for example, details several distinct mesenchymal subpopulations in healthy murine muscle that appear to have specialized cellular roles and behaviors, as evidenced by differential responses to injury stimuli (55). Furthermore, Bensalah et al. demonstrated that human fibroblastic populations can vary greatly between healthy muscles with different ECM content and exhibit considerable behavioral changes in response to disease (56). In the current study, myofibroblasts were investigated using colabeling for the established markers PDFGRα, vimentin, and αSMA, and the clearance of these cells corresponded well with a significant decrease in Postn, also indicative of a reduction in myofibroblasts. It is, however, unclear at this time whether myofibroblasts identified in this manner represent their own distinct mesenchymal (sub)population, a subset of a larger subpopulation, or a heterogeneous group that spans multiple subpopulations. Therefore, it is important to acknowledge that other interstitial cell populations are possibly affected by NOX4 targeting in dystrophic muscle. This is evidenced by the significant reduction in total PDGFRα^+^ cells (Supplemental Figure 4C and Figure 6C), which could represent direct NOX4-mediated effects on these cells and/or be attributed to an indirect mechanism caused by myofibroblast clearance. These points highlight the importance of understanding how muscle fibroblastic populations are affected by chronic disease, as well as how they interact with each other in homeostatic and diseased environments. Additionally, the possibility that nonfibroblastic cell populations found in the muscle interstitium, including macrophages and vascular smooth muscle cells (57, 58), are affected by NOX4 targeting cannot be ruled out at this time.

A critical finding of this work is that the pharmacological treatment with the small-molecule NOX1/4 inhibitor GKT831 (also known as setanaxib) recapitulates much of the phenotype improvements observed with NOX4 ablation. This is important because it demonstrates that the benefits incurred via global NOX4 ablation can be achieved postnatally using pharmacological inhibitors, and, therefore, are not attributable to developmental changes that may have occurred due to NOX4 ablation. GKT831 was chosen for this study because it is a clinical-stage compound with good oral bioavailability and a high safety profile (30); therefore, it has the ability to be rapidly implemented into clinical trials for DMD and/or other MDs. GKT831 previously failed to improve primary outcome measures in a phase II trial for diabetic nephropathy (ClinicalTrials.gov NCT02010242; ref. 30); however, this is likely reflective of the limited regeneration capacity of the kidney. Indeed, a major component of the muscle remodeling identified following GKT831 treatment is improved muscle regeneration (Figure 5). Thus, implementation of GKT831, and/or other NOX4-inhibiting molecules, for the treatment of fibrotic conditions in more regenerative organs, including skeletal muscle, is anticipated to have a more optimistic outcome. While the data of the current study demonstrate NOX4 is an effective therapeutic target in dystrophic muscle by targeting interstitial components of the disease, a dual NOX2/4 inhibitor, such as that recently described by Szekeres et al. (59), may offer additional benefits by also targeting NOX2, which drives dysfunction in muscle fibers (41, 42). Furthermore, such a strategy may offer added cardioprotection, as both NOX2 and NOX4 are capable of contributing to cardiomyocyte dysfunction and cardiac pathology (59–62).

Currently, the landscape of DMD therapeutics is at a pivotal crossroad. Advancements in gene therapy have led to clinical trials evaluating the delivery of miniaturized dystrophin transgenes to the muscles of DMD patients (63). If successful, these therapies will transform DMD into a more slowly progressing disease that still exhibits progressive replacement of muscle with ECM, as occurs in Becker MD patients (64, 65). Furthermore, it is not clear how preexisting disease burden will affect the delivery or efficacy of these gene therapies, particularly in the case of treating older DMD patients. Thus, therapeutics capable of remodeling disease-burdened muscle will remain a major clinical need for the management of DMD, whether as monotherapies or in combination with gene therapy. The current study indicates that NOX4-inhibiting strategies represent effective remodeling therapeutics capable of reducing fibrosis and enhancing muscle regeneration in dystrophic muscle through the targeting of myofibroblasts. While this approach does not correct the primary defect in DMD, namely sarcolemmal instability caused by loss of dystrophin, is does address the major secondary pathologies of failed regeneration and fibrosis; therefore, these findings will likely be broadly impactful for the treatment of both genetic and nongenetic muscle diseases that exhibit progressive fibrosis and failed regeneration.

## Methods

### Animals.

D2.*mdx* (stock number 013141) and D2.WT (stock number 000671) mice used in this study were originally obtained from The Jackson Laboratory. The Nox4^KO^:*mdx* mouse line was created by crossing the *Nox4^tm1Kkr^* knockout allele (ref. 24; The Jackson Laboratory, stock number 022996) onto the D2.*mdx* background for 5 generations, as previously reported (20). Following the second backcross, only *Nox4^tm1kkr/+^* mice homozygous for the DBA/2J polymorphism of *Ltbp4* were selected for continued line development (21). This is because both *Nox4* and *Ltbp4* are located on murine chromosome 7, and crossover is required for both alleles to segregate together. At the completion of the backcrossing, *Nox4* heterozygous breed pairs were mated to produce Nox4^WT^:*mdx* and Nox4^KO^:*mdx* littermates that were used in experiments. All mice were genotyped for *Nox4* using primer sequences provided by The Jackson Laboratory, and for *Ltbp4* and *mdx* alleles using published genotyping primers (21).

Mice were housed 3–5 mice per cage, randomly assigned into groups, provided ad libitum access to food (NIH-31 Open formulation diet; Envigo, 7917), water, and enrichment, and maintained on a 12-hour light/12-hour dark system. Once daily (q.d.) GKT831 (purchased from Ambeed, Inc.) was administered orally (p.o.) suspended at a concentration of 36 mg/mL in a vehicle consisting of sterilized sunflower seed oil (Sigma-Aldrich). This method of drug delivery results in voluntary ingestion of administered solution by mice, similar to the previously reported administration using a syrup-based vehicle (18). The GKT831 treatment group received a dose of 60 mg/kg, which yields favorable drug exposure in mice (22). Three-month treatment regimens were initiated at 3 months of age, whereas acute, 7-day treatment regimens were initiated at 6 months of age.

### Immunofluorescence and histological evaluations.

OCT-embedded murine tissues were cryosectioned at 10 μm and fixed in 4% paraformaldehyde (PFA). Immunofluorescence analysis was performed using anti-NOX4 (1:2000; Abcam, 13303), anti-laminin (1:800; Novus, MAB2549), anti-vimentin (1:1000; Novus, MB300), anti-PDGFRα (1:500; R&D Systems, AF1062), anti-αSMA (rabbit polyclonal; 1:1000; Abcam, ab5694), anti-αSMA (mouse monoclonal; 1:1000; Abcam, 7817), anti–4-HNE (1:200; Invitrogen, MA5-27570), anti-utrophin (1:100; Vector Labs, VP-U579), anti-CD31 (1:200; Bio-Rad, MCA2388), anti–α-actinin 2 (1:2000; Sigma-Aldrich, A7811), anti–myosin heavy chain IIA (1:100; DSHB, SC-71), anti–myosin heavy chain I (1:100; DSHB, BA-F8), anti-fibronectin (1:2000; Sigma-Aldrich, F7387), anti–Sca-1 (1:250; BioLegend, 160902), anti-Postn (1:500; Abcam, ab14041), anti-Pax7 (1:100; R&D Systems, MAB1675), anti–myosin X (1:800; Sigma-Aldrich, HPA024223), and anti–active caspase-3 (1:1000; Cell Signaling Technology, 9661) primary antibodies. Mouse tissue sections incubated with mouse monoclonal antibodies were first incubated with a solution containing donkey anti–mouse IgG AffiniPure Fab fragments (diluted 1:25 in PBS; Jackson ImmunoResearch, 715-007-003) for 1 hour prior to blocking. Following overnight incubation in primary antibody, sections were rinsed with PBS and incubated with the appropriate species/isotype-specific secondary antibody (1:500 dilution; Molecular Probes). Appropriate isotype controls were used to verify specificity of signal to primary antibody labeling. Lipofuscin-dependent autofluorescence was quenched using a 0.1% solution of Sudan black B (Sigma-Aldrich) following secondary antibody incubation. Slides were coverslipped using Prolong Gold mounting reagent (Invitrogen). Images were acquired using a Leica SP8 confocal microscope in sequential scan mode. Human samples were obtained through the National Disease Research Interchange. Picrosirius red staining was performed as previously described (19) following decalcification of muscle sections using Formical-2000 (StatLab). Slides were visualized with a Leica DMR microscope, and images were acquired using a Leica DFC310FX camera interfaced with Leica LAS X software. In all imaging experiments, comparative images were stained simultaneously and acquired using identical settings. Images were processed and analyzed by investigators blinded to study groups using ImageJ software (NIH), as previously described (19). Image quantification for each sample consisted of the mean value from 5 independent and randomly selected fields of view for each muscle section.

### Assessment of muscle function.

Functional assessments of the EDL and diaphragm muscles were performed as previously described (18) by the University of Florida Physiological Assessment Core. Muscles of anesthetized mice were dissected and placed in physiological Ringer’s solution gas equilibrated with 95% O_2_/5% CO_2_. After determining optimal length, muscles were subjected to 3 isometric contractions (stimulated at 120 Hz for 500 ms) to determine maximal tetanic tension (Po). Following these procedures, muscles were weighed, frozen embedded in OCT or snap-frozen, and stored at –80°C until further use.

### Collagen assay.

Snap-frozen gastrocnemius samples were pulverized and transferred to preweighed 2 mL microcentrifuge tubes. Tendon pieces were removed from samples during the pulverization procedure. Deionized H_2_O was added to the sample at a volume of 10-times the sample mass, and the tissues were vigorously disrupted using a hand homogenizer (Benchmark Scientific Model D1000). A volume of 200 μL for each resulting sample homogenate was transferred to preweighed screw-top microcentrifuge tubes, and samples were completely desiccated by overnight incubation at 65°C in order to determine the dry mass of the sample to be assayed. This is important because of potential differences in tissue mass caused by edema that may affect assay results. Following tissue dry mass determination, the collagen content of the samples was measured using a colorimetric Total Collagen Assay kit (Biovision, K218), following the manufacturer’s directions in a 96-well plate. Assay data were collected using a SpectraMax i3x multimode spectrophotometer (Molecular Devices) at a wavelength of 560 nm.

### Gene expression.

Gene expression analysis was conducted as previously described (18) using the following mouse-specific primers: *Col1a2* (forward) 5′-ATGGTGGCAGCCAGTTTGAA-3′ and (reverse) 5′-TCCAGGTACGCAATGCTGTT-3′; *Acta2* (forward) 5′-CTACTGCCGAGCGTGAGATTGTCC-3′ and (reverse) 5′-GAGGGCCCAGCTTCGTCGTATT-3′; *Il6* (forward) 5′-AACCACGGCCTTCCCTACTTC-3′ and (reverse) 5′-TCTGGCTTTGTCTTTCTTGTTATC-3′; *Tgfb1* (forward) 5′-GACTCTCCACCTGCAAGACCAT-3′ and (reverse) 5′-GGGACTGGCGAGCCTTAGTT-3′; *Gapdh* (forward) 5′-AGCAGGCATCTGAGGGCCCA-3′ and (reverse) 5′-TGTTGGGGGCCGAGTTGGGA-3′. Relative gene expression quantification was performed using the ΔΔCt method with *Gapdh* as the normalization gene.

### Immunoblotting.

Immunoblotting was performed as previously described (18) using the following primary antibodies: anti-NOX4 (1:2000; Abcam, 13303), anti-fibronectin (1:2000; Sigma-Aldrich, F7387), anti-SDHA (1:2000; Invitrogen, 459200), and anti-HSP60 (1:4000; Abcam, ab8071). Quantified band signal intensities were measured using Image Studio Lite software (LI-COR Biosciences), normalized to Ponceau red–visualized loading, and reported relative to respective control samples.

### Cell isolation.

Gastrocnemius muscles were dissected using aseptic technique, cleared of visible tendons and connective tissue, weighed, rinsed in PBS containing 1% Pen/Strep (Gibco, 15140122), and finely minced into a uniform slurry using approximately 0.25 mL of a filter-sterilized digest media containing DMEM (Gibco, 10566024), 1% Pen/Strep, 50 mg/mL collagenase IV (Worthington, LS004188), and 6 U/mL Dispase (Gibco, 17105041). The slurry was diluted to a 10:1 medium/muscle mass ratio and incubated at 37°C for 1 hour with constant agitation. The muscle digest mix was neutralized with cold isolation media containing DMEM, 1% Pen/Strep, and 10% FBS (Gibco, A3840002), triturated, and filtered through a 40 μm nylon filter (Falcon, 352340). Following centrifugation, resuspension, and cell counting, cells were subjected to magnetic-activated cell sorting (MACS) using the CD140a/PDGFRα MicroBead Kit (Miltenyi Biotec, 130-101-502), following the manufacturer’s directions. Purified PDGFRα^+^ cells were centrifuged, resuspended in growth media containing phenol red–free DMEM (Gibco, 31053028), 10% FBS, 1% Pen/Strep, and 4 mM L-glutamine (Gibco, A2916801), quantified, and plated onto either gelatin-coated coverslips for immunofluorescence (after 16 hours of incubation) or gelatin-coated Softslip 8 kPa hydrogels (Matrigen, SS24-EC-8) for myofibroblast persistence assays.

### Myofibroblast persistence assay.

Isolated PDGFRα^+^ cells were plated at a density of 2,000 cells/well onto Softslip hydrogels in a 24-well format. After 2 hours, the cells were switched to a modified growth media containing 1% FBS and were incubated for 72 hours. At the endpoint, cells were rinsed with PBS, fixed with 4% PFA for 30 minutes, permeabilized with 0.1% Triton X-100 in PBS, and subjected to immunofluorescence procedures for PDGRFα and αSMA staining (see above). The entire hydrogel area for each sample was acquired using tile scan mode. Persistent myofibroblasts were quantified as the percentage of αSMA^+^ cells identified on the hydrogels after the 3-day incubation period. A minimum of 4 hydrogel replicates were used for each mouse gastrocnemius pair, and the mean of the replicates was used as the value for each mouse used for this experiment (*n* = 6 mice per group).

### Statistics.

Statistical analysis was performed using an unpaired, 2-tailed Welch’s *t* test (α = 0.05; effect size reported as Cohen’s *d*) or 1-way ANOVA (Tukey’s post hoc test; α = 0.05; effect size reports as *η*^2^). A *P* value of less than 0.05 was considered significant. Values are displayed as box-and-whisker plots (boxes represent second and third quartiles, while the whiskers represent the minimum and maximum values; no outliers were excluded from these analyses).

### Study approval.

All animal procedures were approved and conducted in accordance with the University of Florida IACUC and reported using the recommendations of ARRIVE guidelines.

## Author contributions

DWH contributed to all authorship aspects of this work.

## Supplementary Material

Supplemental data

## Version 1 10/24/2022

Electronic publication
