# Supplementary material for: NOX4 inhibition promotes the remodeling of dystrophic muscle
Source: JCI Insight. 2022 Oct 24;7(20):e158316. doi: 10.1172/jci.insight.158316 (PMC9714779; doi:10.1172/jci.insight.158316)
Supplement: Supplemental data [file jciinsight-7-158316-s032.pdf]

SUPPLEMENTAL FIGURES

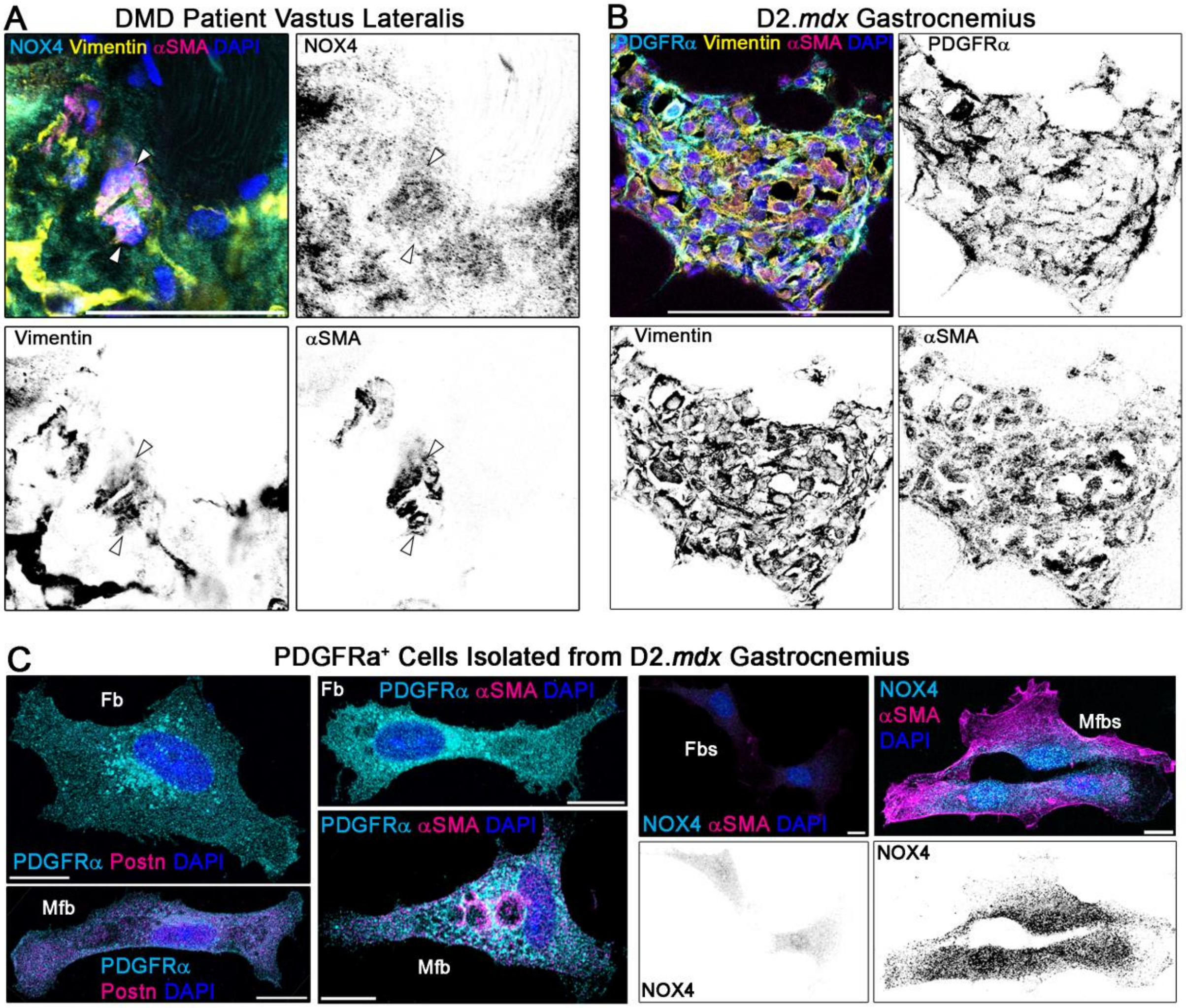

Figure S1. NOX4 expression in myofibroblasts. Related to Figures 1-2. (**A**) Immunofluorescent (IF) detection of NOX4 in SMA$^+$ vimentin$^+$ cells in the interstitium of DMD patient muscle. (**B**) Myofibroblasts in D2.*mdx* gastrocnemius muscles are identified using antibodies against PDGFRα, vimentin, and SMA. (**C**) PDGFRα$^+$ cells were isolated from 6 mo D2.*mdx* muscle and fixed 16 hours following plating. IF analysis reveals fibroblasts (Fbs) and myofiroblasts (Mfbs) are distinguishable by differential staining for Postn and SMA. Amongst these populations, NOX4 is enriched in Mfbs. Scale bars represent (**A-B**) 50 µm or (**C**) 10 µm.

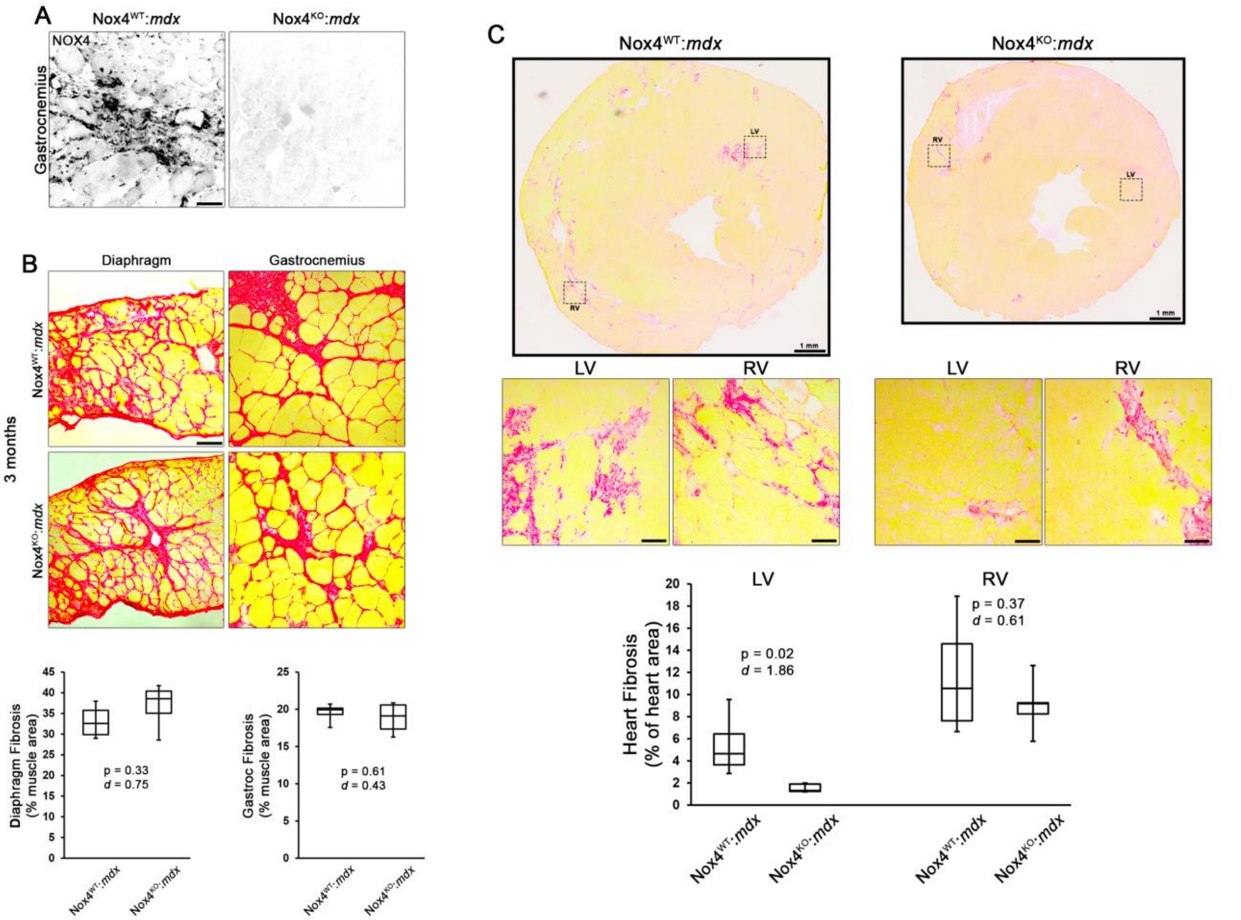

Figure S2. NOX4 ablation does not affect early time course of muscle fibrosis and reduces left ventricular fibrosis. Related to Figure 3. (**A**) Representative immunofluorescent images from Nox4^{WT}:*mdx* and Nox4^{KO}:*mdx* gastrocnemius (gastroc) muscles stained with anti-NOX4 antibody. (**B**) Muscle fibrosis was histologically assessed in the diaphragm and gastroc muscles from 3 month-old Nox4^{WT}:*mdx* and Nox4^{KO}:*mdx* mice using picrosirius red staining (n = 4), revealing no significant differences in muscle fibrosis between the groups at this age. (**C**) Picrosirius red staining reveals reduced left ventricle fibrosis in the hearts of 6 month-old Nox4^{KO}:*mdx* mice. Data are presented as box-and-whisker plots with error bars representing minimum and maximum values. Data were analyzed using unpaired, two-tailed Welch's T-tests ($\alpha = 0.05$; effect size is presented as Cohen's *d*). Scale bars represent 100 µm, unless otherwise noted.

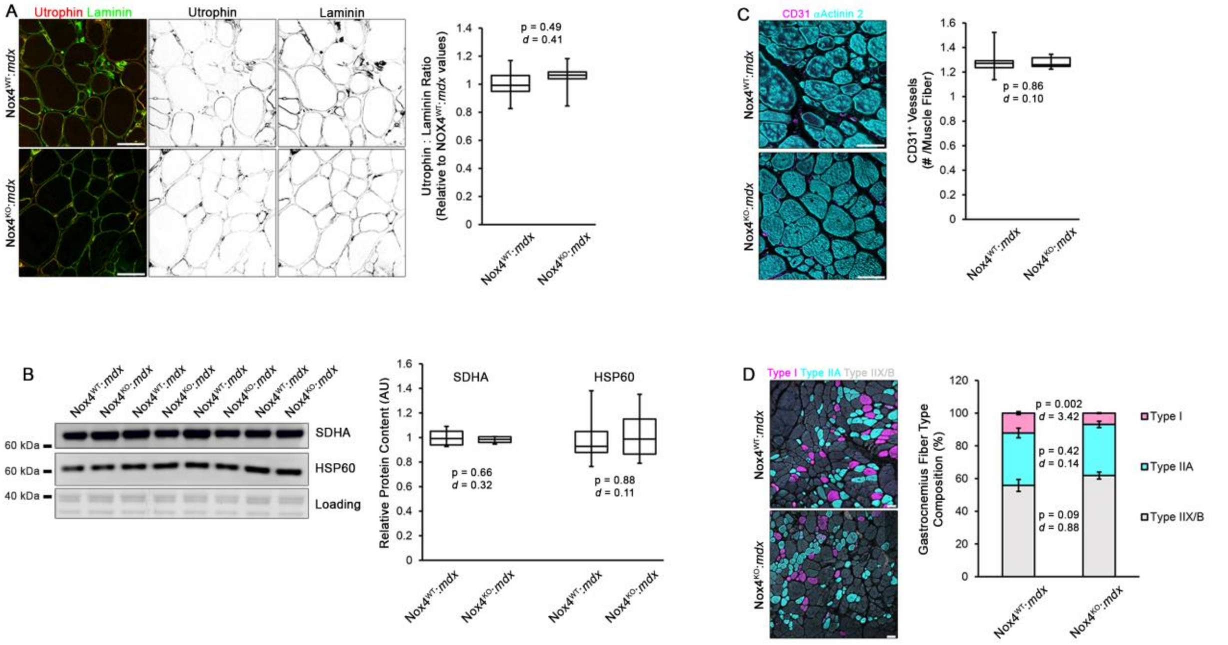

Figure S3. NOX4 ablation does not affect markers of myofiber protection, mitochondrial content, or vascularity. Related to Figure 3. (**A**) Sarcolemmal utrophin content was quantified in Nox4^{WT}:*mdx* and Nox4^{KO}:*mdx* gastrocnemius muscles (n = 6) using line scans across sarcolemmal boundaries and normalizing utrophin signal intensity to the laminin signal. Regions of muscle with apparent regeneration were avoided, as utrophin content is increased in regenerating myofibers and these groups exhibit differential regeneration (**Figure 5**). (**B**) The mitochondrial markers succinate dehydrogenase A (SDHA) and heat shock protein 60 (HSP60) were evaluated via immunoblotting using quadriceps lysates (n = 4). Band intensities were normalized to Ponceau Red-visualized loading content. (**C**) Blood vessel density was quantified in gastrocnemius muscles using the endothelial cell marker CD31 and reported relative to muscle fibers delineated by αActinin 2 staining (n = 6). (**D**) Muscle fiber types were evaluated in gastrocnemius muscles and displayed as the percentage of composition (n = 5-6). Data are presented as (**A-C**) box-and-whisker plots with error bars representing minimum and maximum values or as (**D**) mean ± SEM. Data were analyzed using unpaired, two-tailed Welch's T-tests (α = 0.05; effect size is presented as Cohen's *d*). Scale bars represent 50 µm.

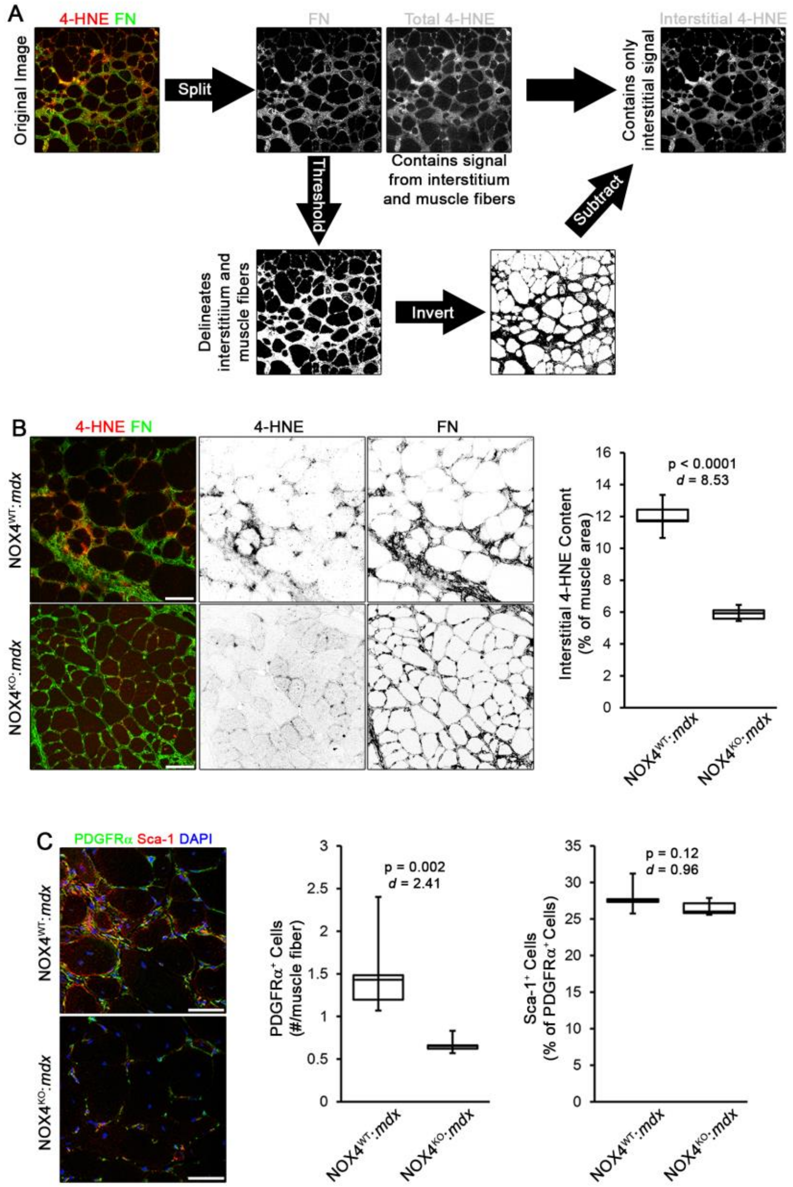

Figure S4. Interstitial lipid peroxidation and FAP numbers decline with NOX4 ablation. Related to Figures 3-4. (**A**) A methodology to specifically quantify interstitial staining of 4-hydroxynonenal (4-HNE) was developed, where fibronectin (FN) co-staining is used to delineate interstitial and myofiber areas. The FN signal is thresholded, inverted, and subtracted from the 4-HNE signal to specifically visualize the interstitial component of 4-HNE labeling. (**B**) This methodology was applied to analyze interstitial 4-HNE content in the gastrocnemius muscles of Nox4^{WT}:*mdx* and Nox4^{KO}:*mdx* mice and reported as percent of muscle area (n = 6). Representative images include total 4-HNE signal to show myofiber-specific lipid peroxidation occurs in NOX4 ablated *mdx* mice. (**C**) PDGFRα and Sca-1 staining were used to quantify FAPs in the gastrocnemius muscles of Nox4^{WT}:*mdx* and Nox4^{KO}:*mdx* mice (n = 6). Data are presented as box-and-whisker plots with error bars representing minimum and maximum values. Data were analyzed using unpaired, two-tailed Welch's T-tests (α = 0.05; effect size is presented as Cohen's *d*). Scale bars represent 50 µm.
